# Polyamines Counteract Carbonate-Driven Proteasome Stalling in Alkaline Conditions

**DOI:** 10.3390/biom10121597

**Published:** 2020-11-24

**Authors:** Anna A. Kudriaeva, George A. Saratov, Alena N. Kaminskaya, Vasiliy I. Vladimirov, Petro Yu Barzilovich, Alexey A. Belogurov

**Affiliations:** 1 Shemyakin-Ovchinnikov Institute of Bioorganic Chemistry, Russian Academy of Sciences, 117997 Moscow, Russia; anna.kudriaeva@gmail.com (A.A.K.); saratovgosha@gmail.com (G.A.S.); kaminskayaan@mail.ru (A.N.K.); pjetro@yandex.ru (P.Y.B.); 2 Phystech School of Biological and Medical Physics, Moscow Institute of Physics and Technology (National Research University), 141701 Dolgoprudny, Russia; 3 Pushchino Branch of Shemyakin-Ovchinnikov Institute of Bioorganic Chemistry, Russian Academy of Sciences, 142290 Pushchino, Russia; vasiliy.i.vladimirov@gmail.com; 4 Faculty of Fundamental Medicine, Lomonosov Moscow State University, 119991 Moscow, Russia

**Keywords:** proteasome, polyamine, spermine, intracellular alkalization, activation, carbonate, inhibition

## Abstract

Cancer cells tend to increase intracellular pH and, at the same time, are known to intensively produce and uptake polyamines such as spermine. Here, we show that various amines, including biogenic polyamines, boost the activity of proteasomes in a dose-dependent manner. Proteasome activity in the classical amine-containing buffers, such as 2-(N-morpholino)ethanesulfonic acid (MES), Tris, (4-(2-hydroxyethyl)-1-piperazineethanesulfonic acid (HEPES), glycylglycine, bis-Tris propane, and bicine, has a skewed distribution with a maximum at pH of 7.0–8.0. The activity of proteasomes in buffers containing imidazole and bis-Tris is maintained almost on the same level, in the pH range of 6.5–8.5. The third type of activation is observed in buffers based on the amino acids arginine and ornithine, as well as the natural polyamines spermine and spermidine. Proteasome activity in these buffers is dramatically increased at pH values greater than 7.5. Anionic buffers such as phosphate or carbonate, in contrast, inhibit proteasome activity during alkalization. Importantly, supplementation of a carbonate–phosphate buffer with spermine counteracts carbonate-driven proteasome stalling in alkaline conditions, predicting an additional physiological role of polyamines in maintaining the metabolism and survival of cancer cells.

## 1. Introduction

Intracellular pH adjustment plays a crucial role in the metabolism and survival of the mammalian cell, as the activity of the majority of enzymes significantly depends on its value [1]. Essentially, cells from all types of mammalian tissues produce acid because of CO_2_ and lactic acid, generated by mitochondrial respiration and fermentative metabolism, respectively [2,3]. For almost a century, it has been known that cancer cells reorganize their metabolism in accord with the Warburg effect [4,5]. Oxygen depletion, together with genetic and epigenetic changes [6], shifts the metabolism of the cancer cells toward a more glycolytic phenotype [7], characterized by an exacerbated output of lactic acid [2]. Lactic acidosis, a high-lactate concentration with an acidic intracellular pH, significantly enhances the survival of cancer cells under a lack of glucose. G1/G0 phase arrest, induction of autophagy, and inhibition of apoptosis are directly associated with lactic acidosis-mediated resistance to glucose deprivation. The high-lactate concentration, lactosis, with weak basic pH, has a pronounced effect on cell survival during glucose starvation [8].

Acidification of the extracellular milieu and concomitant intracellular alkalization of the cytoplasm are highly common for tumor cells [9]. Extracellular pH in tumors is typically lower (6.5–6.9) than in normal tissue (7.2–7.5) [10]. Activation of various plasma membrane transporters and acid efflux proteins that control pH homeostasis [11], including monocarboxylate transporters, carbonic anhydrases, and Na^+^–H^+^ exchangers, promotes intracellular alkalization [12]. This reverse pH gradient is associated with tumor aggressiveness, i.e., proliferation, invasion, metastasis, and treatment resistance [2,9,13,14,15,16]. Alkalization of the tumor’s extracellular pH inhibits carcinogenesis [15], whereas mathematical modeling suggests that systemic buffers [17] with pK*a* 7.0 [18] may be used in order to accomplish such a pH shift. It is expected that bicarbonate shifts the intratumoral metabolism from lactic acidosis to lactosis and thus inhibits tumor growth and enhances the necrosis of cancer cells [19].

The natural polyamines spermidine and spermine are polycations with three and four amine groups, respectively. Almost every living cell contains polyamines in up to millimolar concentrations [20]. Rapidly growing cells activate the synthesis of polyamines from arginine and s-adenosylmethionine; spermine and spermidine are formed from 1,4-diaminobutane (putrescine). The biosynthesis of putrescine may be driven via classical and alternative pathways. In the classical pathway, urea and carbon dioxide are removed from arginine by arginase and ornithine decarboxylase (ODC), while the biochemistry of the alternative pathway includes the removal of carbon dioxide by arginine decarboxylase and, further, removal of urea by agmatinase [21]. Polyamines are involved in the crucial cellular processes closely linked to cell growth and differentiation, such as DNA synthesis and stability, regulation of transcription, protein phosphorylation, and ion channel regulation [22,23,24]. The concentration of polyamines, as well as the gene expression and activity of the enzymes involved in polyamine biosynthesis, especially ODC, is higher in tumors in comparison with the normal surrounding tissues [25,26,27,28,29]. Polyamine levels are increased in the blood and urine of patients with cancer and, in addition, the concentration of polyamines is positively correlated with poor prognosis [30].

Previously, it was shown that polyamines may increase the activity of enzymes, such as α-chymotrypsin [31], and act like chemical chaperones [32]. In contrast, proteins enriched with basic amino acids such as arginine and lysine may directly bind proteasomes [33,34] and are capable of further translocation into the proteolytic chamber [35,36]. Here, we investigate how the alkalization and increased concentration of polyamines may modulate the activity of proteasome, a part of the ubiquitin proteasome system (UPS), which specifically degrades thousands of intracellular proteins. The UPS consists of hundreds of ubiquitin ligases [37], conjugating the small protein ubiquitin with a substrate, which is further recognized and degraded by proteasome particles [38]. Proteasomes are absolutely necessary for cell functioning; moreover, their activity follows the overall cell metabolism. Inhibition of proteasomes in rapidly growing tumor cells is regarded as an effective therapeutic intervention during cancer [39]. Several classes of small molecules, including denaturing reagents (e.g., SDS), lipids, and peptides, namely those with the HbYX motif, were shown to activate the proteasome at relatively high concentrations (reviewed in [40]). Previously, it was reported that polylysine at 100 μM [41] and other polycationic substances, including polyarginine, protamine, and histone H1 [42], activated the 20S proteasome and the bacterial ATP-dependent protease Hs1VU [43]. Herein, we show that polyamines, in contrast to anionic buffers, significantly increase proteasome activity in a pH- and concentration-dependent manner in vitro. This observation may be a step forward in the understanding of the interplay among polyamines, proteasomes, and the carbonate-driven alkalization of cancer cells observed in vivo.

## 2. Materials and Methods

### 2.1. Materials

Fluorogenic proteasome substrates were purchased from UBPbio (Aurora, IL, USA). The other solvents and chemicals were of reagent grade and acquired from either Merck KGaA (Darmstadt, Germany) or Helicon (Moscow, Russian Federation).

### 2.2. Purification of Proteasomes from Bovine Liver

The proteasome samples were prepared according to the protocol described in [33]. A bovine liver was mechanically homogenized in a hypotonic lysis buffer containing 10 mM Tris-HCl (pH 7.9), 1.5 mM MgCl_2_, 1 mM ATP, and 10 mM KCl. Furthermore, 2.0 mM DTT, 0.025% digitonin (MilliporeSigma, Burlington, MA, USA), 1.0 mM phenylmethylsufonyl fluoride, and 0.1% *N*-dodecyl b-D-maltoside were added. The prepared liver homogenate was subjected to 20 cycles of high-pressure homogenization (10 cycles at 350 bar and 10 cycles at 1000 bar, with an APV 2000 homogenizer (SPX Flow, Charlotte, NC, USA)) and further incubated for 30 min on ice. Cell debris was removed by centrifugation at 30,000× *g* for 30 min at 4 °C. The S30 cytoplasmic extract was supplemented with a purification buffer to 1× concentration (50 mM Tris-HCl (pH 7.0), 50 mM KCl, 10 mM MgCl_2_, 1 mM ATP, and 10 mM β-glycerophosphate) from a 10× stock, followed by the addition of sucrose powder at a concentration of 20% (*w*/*v*). The extract was incubated at room temperature on a magnetic stirrer for 1 h and further centrifuged at 30,000× *g* for 30 min at 4 °C. The clarified extract was subjected to differential precipitation with polyethylene glycol (PEG) with a mean molecular weight (MW) 400 (PEG400). PEG400 was added at a concentration of 20% (*v*/*v*) to the extract under stirring at 4 °C and then incubated for 20 min. The precipitated proteins were centrifuged at 30,000× *g* for 30 min at 4 °C. The supernatant was then precipitated by raising the concentration of the PEG400 to 30% (*v*/*v*) as described above. The precipitate, which contained the 26S and 20S proteasomes, was recovered by centrifugation at 30,000× *g* for 30 min at 4 °C. The pellet was resuspended in a buffer for ion-exchange chromatography that contained 10 mM Tris-HCl (pH 7.5), 200 mM NaCl, 1 mM ethylenediaminetetraacetic acid (EDTA), 1 mM dithiothreitol (DTT), 1 mM ATP, and 10% glycerol. After centrifugation at 30,000× *g* for 30 min at 4 °C to remove the insoluble material, the extracts were subjected to ion-exchange chromatography utilizing Q-sepharose resin (with a 200–600 mM NaCl gradient). Fractions containing 20S and/or 26S proteasomes were identified according to the rate of Suc-Leu-Leu-Val-Tyr-aminomethylcoumarin (Suc-LLVY-AMC) hydrolysis in the presence or absence of 0.02% SDS. The selected fractions were pooled and precipitated by the addition of 40% (*v*/*v*) PEG400. Purified 26S and 20S proteasomes were reconstituted in a buffer containing 25 mM Tris-HCl (pH 7.5), 1 mM DTT, 5 mM MgCl_2_, 1 mM ATP, and 10% glycerol.

### 2.3. Measurement of the Peptidase Activity of Proteasomes

The peptidase activity was determined with 0.5 µg 20S or 0.15 µg 26S proteasome incubated with 20 μM of the fluorogenic substrate Suc-LLVY-AMC, Ac-Arg-Leu-Arg-aminomethylcoumarin (Ac-RLR-AMC), or Ac-Gly-Pro-Leu-Asp-aminomethylcoumarin (Ac-GPLD-AMC) (excitation wavelength of 380 nm and an emission wavelength of 440 nm) in a volume of 100 µL by a microplate reader (Varioscan Flash, Thermo Fisher Scientific, Waltham, MA, USA) at 37 °C. The buffer used for measurement of the activity of the proteasomes contained 25 mM of an appropriate buffer at various pH levels, 1 mM ATP, 1 mM DTT, and 5 mM MgCl_2_. The pH of all buffers was adjusted at 37 °C, similar to the temperature used during the measurement of proteasome activity.

### 2.4. In Vitro Ubiquitination and Proteasome Hydrolysis

In Vitro ubiquitination was performed as follows: E1 (2 μg) (Addgene #63571) obtained according to [44] was mixed with UbcH5c (4 μg) (Addgene #12643) obtained according to [45], ubiquitin (20 μg), and Ub-TagGFP2 (4 μg). The pET22-based plasmid coding for the Ub-TagGFP2-His_6_ was generated by the overlap PCR utilizing the pTagGFP2-C vector (Evrogen, Moscow, Russia) as a matrix. Two terminal glycine residues in the ubiquitin sequence were substituted by valine in order to enhance resistance toward deubiquitination enzymes. The Ub-TagGFP2 was expressed in *Escherichia coli* (BL21(DE3) strain) and further purified by immobilized metal affinity chromatography (IMAC). The reaction was incubated at 37 °C overnight in a buffer containing 20 mM phosphate buffer (pH 8.0), 100 mM NaCl, 5 mM MgCl_2_, 3 mM ATP, and 1 mM DTT. The final volume of the reaction was 65 μL. Furthermore, 5 μL from the in vitro ubiquitination reaction was supplemented with a final concentration of 20 mM phosphate buffer (pH 7.5 or 8.5), 100 mM NaCl, 5 mM MgCl_2_, 3 mM ATP, 1 mM DTT, purified 26S proteasome (10 μg), and spermine (10 mM). The reaction volume was adjusted to 20 μL. The reaction was incubated at 37 °C overnight.

### 2.5. Data Analyses

Statistical analyses were performed with SigmaPlot software (Systat Software, San Jose, CA, USA), utilizing unpaired *t*-tests. Any *p*-values < 0.05 were taken as significant. The data were fitted to a polynomial square of an exponential or Gauss function.

## 3. Results and Discussion

### 3.1. Four Types of pH-Dependent Proteasome Activation by Amines

**Figure 1:**
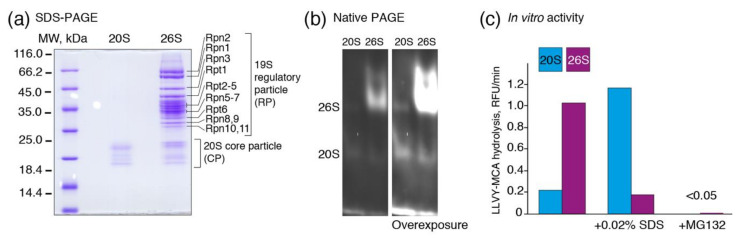
(**a**) The purified 20S (1.5 μg) and 26S (5.0 μg) proteasomes were subjected to polyacrylamide gel electrophoresis and further stained with Coomassie blue R250. Subunits of the 19S regulatory particle and the molecular weight marker are indicated. (**b**,**c**) The activity of 20S and 26S proteasomes was analyzed by native PAGE further saturated with Suc-LLVY-AMC (**b**) or measured in the presence or absence of 0.02% SDS and the proteasome inhibitor MG132 (**c**).

Proteasomes may exist in two basic states, namely as a core particle (20S) or as 20S capped with a 19S regulatory particle (26S). Purified samples of the 20S and 26S proteasomes from the bovine liver were analyzed by polyacrylamide gel electrophoresis in the denaturing (Figure 1a) and native (Figure 1b) conditions. Activity of the 26S proteasome samples was inhibited in the presence of 0.02% SDS; both proteasomes were completely inhibited in presence of specific inhibitor MG132 (Figure 1c). The 20S proteolytic core has chymotryptic-, tryptic-, and caspase-like activities; the former is regarded as the most crucial for protein degradation [46]. We therefore firstly measured the chymotryptic activity of the purified bovine 20S and 26S proteasomes at a pH range of 6.5–8.5 in the various (poly)amine buffer systems. In order to estimate the direct effect of amines on proteasomes, the pH in the buffer system was maintained by the same amine.

**Figure 2:**
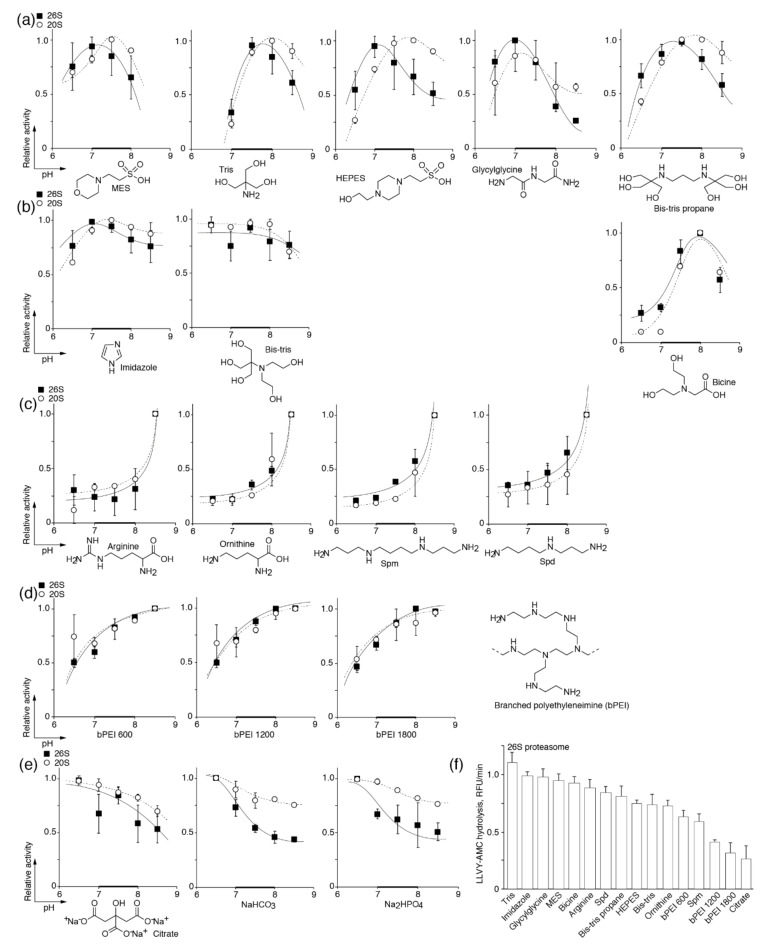
The pH dependence of the proteasomal chymotrypsin-like peptidase activity in different buffers containing (poly)amines (**a**–**d**) and anionic buffers (**e**). The skewed distribution (**a**), pH-independent (**b**), bursting (**c**) and progressive (**d**) types of activation are shown. Activity levels of the 26S (■) and 20S (○) proteasomes were measured using succinyl-Leu-Leu-Val-Tyr-7-amido-4-methylcoumarin (Suc-LLVY-AMC) at 25 mM of each buffer system. Relative activity was calculated as the ratio of activity at a distinct pH to the maximal activity within the tested pH range. (**f**) Absolute activity of the 26S proteasome in different buffers at the optimal pH value for each buffer system. The data represent the average and standard deviation (error bars) from four independent measurements. Natural polyamines (Spm, Spd) and branched polyamines (bPEI) were used at a concentration of 5 mM; all other buffers were at 25 mM. The data were fitted to a polynomial square of an exponential or Gauss function. The physiologically relevant pH range is shown by a bold line.

The profile of proteasome activity in the classical amine-containing buffers such as MES, Tris, HEPES, glycylglycine, bicine, and bis-Tris propane has a skewed distribution with a maximum at pH of 7.0–7.5 (Figure 2a). Meanwhile, buffers containing imidazole and bis-Tris maintained proteasome activity almost on the same level within the pH range studied (Figure 2b). The third type of activation was observed in the buffers based on the amino acids arginine and ornithine and the natural polyamines spermine (Spm) and spermidine (Spd). The activity of proteasomes in these buffers was dramatically increased at pH values greater than 8.0 (Figure 2c). Additionally, we studied synthetic branched polyamines (bPEI) with a molecular weight ranging from 0.6 to 1.8 kDa. These buffer systems revealed a fourth type of activation, which is characterized by a prolonged activity curve with saturation at a pH level >8.0 (Figure 2d). There was no evident correlation of activation type with either the presence of a primary, secondary, or tertiary amine group; nonetheless, the activity of proteasomes reached its maximum near the pK*a* value of the buffer system. The activity profile of the 20S proteasome in almost all buffers followed that of 26S. In the case of a “skewed distribution”, the maximum activity of the 20S proteasome was shifted by half of a pH unit toward the alkaline area (Figure 2a).

The anionic buffer systems, such as citrate, carbonate, and phosphate, inhibited the activity of the proteasomes at an increased pH (Figure 2e). The activity of the 20S proteasome was less affected by an increase in the pH value in the anionic buffers in comparison with the 26S proteasome, which means that inhibition of 26S may occur due to the rearrangement of the 19S subparticle or partial dissociation of the 26S proteasome to 20S and 19S. The absolute activity of the 26S proteasome in different buffers at the optimal pH value for each buffer system is shown in Figure 2f. As the 26S and 20S proteasomes are more or less similarly affected by amines and the pH value, we suggest that the activation of proteasomes is rather caused by their influence on the catalytic core particle.

**Figure 3:**
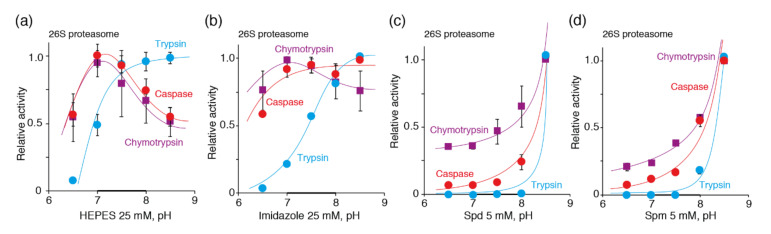
The pH dependence of the proteasomal chymotrypsin-like (violet), trypsin-like (blue), and caspase-like (red) peptidase activities in the buffers containing HEPES (**a**), imidazole (**b**), spermidine (**c**), and spermine (**d**). Activity of the 26S proteasome was measured using Suc-LLVY-AMC (chymotrypsin-like), Ac-RLR-AMC (trypsin-like) or Ac-GPLD-AMC (caspase-like) fluorogenic substrates at 25 mM of the HEPES and imidazole buffer systems and 5 mM of the spermidine and spermine buffer systems. Relative activity was calculated as the ratio of activity at a distinct pH to the maximal activity within the tested pH range. The data represent the average and standard deviation (error bars) from four independent measurements. The data were fitted to a polynomial square of an exponential or Gauss function. The physiologically relevant pH range is shown by a bold line.

Previously, it was shown that histone H3 significantly enhances 20S-mediated degradation of the oxidized B-chain of insulin in terms of the cleaving bonds, mainly after acidic and branched chain amino acids, i.e., positive allosteric activation of the caspase- and chymotrypsin- but not trypsin-like activities [47]. Indeed, measurement of the caspase- and trypsin-like activities of proteasomes in the buffers based on HEPES, imidazole, and the natural polyamines Spm and Spd at different pH levels (Figure 3) revealed that the caspase-like activity of the 26S proteasome follows the profile of the chymotrypsin-like activity in all buffers. The trypsin-like activity was increased, similar to the caspase- and chymotrypsin-like activities in the Spm- and Spd-based buffers. In the HEPES and imidazole buffers, the trypsin-like activity of proteasomes has its own profile, with saturation at pH values more than 7.5–8.0, as was reported previously [48].

### 3.2. Polyamines Counteract Carbonate-Driven Proteasome Stalling in Alkaline Conditions

**Figure 4:**
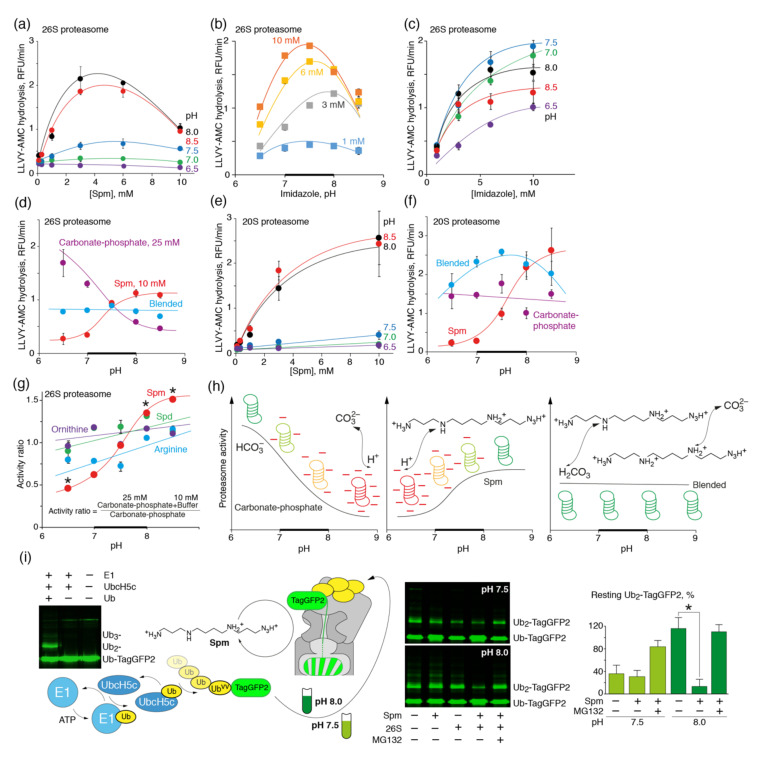
Spermine enhances the proteasomal chymotrypsin-like peptidase activity in a pH-dependent manner and counteracts carbonate-driven proteasome stalling at an alkaline pH. Chymotrypsin-like activity of the 26S (**a**,**d**) or 20S (**e**,**f**) proteasomes was measured at different pH and Spm concentrations (**a**,**e**) or at different pH in the amine buffer systems (**d**,**f**) containing 25 mM carbonate–phosphate (24:1) (violet), 10 mM Spm (red) or their mixture (cyan). (**b**,**c**) The pH dependence of the proteasomal chymotrypsin-like peptidase activity in the buffers containing various concentrations of imidazole at different pH. Activity of the 26S proteasome was measured using Suc-LLVY-AMC at the indicated concentrations of imidazole and pH values. The data represent the average and standard deviation (error bars) from four independent measurements. (**g**) Ratio of the chymotrypsin-like activity of the 26S proteasome measured in the 25 mM carbonate–phosphate buffer supplemented with 10 mM arginine (blue), ornithine (violet), Spd (green) or Spm (red) to the activity of the 26S proteasome measured in the 25 mM carbonate–phosphate buffer. Asterisks denote a statistically significant difference. The data were fitted to a polynomial square of an exponential or Gauss function and are shown as means ± SD (error bars) from triplicate determinations. (**h**) Deprotonation of the 26S proteasome by carbonate ions at an alkaline pH or by Spm at a neutral pH leads to proteasome inhibition. Simultaneous exposure of the 26S proteasome to Spm and carbonate results in proton transfer between bicarbonate and Spm at a neutral pH and protonation of carbonate ions by protons from Spm at an alkaline pH. The presence of Spm as a proton donor at alkaline pH protects the proteasome and thus preserves its activity. (**i**) Ub-TagGFP2 was ubiquitinated in vitro by a reconstituted E1-UbcH5c cascade and further mixed with the 26S proteasome at various pH levels in the presence or absence of Spm. Bars on the right represent the percentage of the di-ubiquitinated TagGFP2 hydrolyzed by the 26S proteasome compared with the control sample. Standard deviations are shown. The physiologically relevant pH range is shown by a bold line.

We next investigated if polyamines, like Spm, may increase the activity of proteasomes in the presence of carbonate. To this end, we firstly tested various concentrations of Spm at different pH levels, ranging from 6.5 to 8.5 (Figure 4a). Maximal activity of the26S proteasome was observed at a Spm concentration of 3 mM and a pH of 8.0–8.5. Interestingly, a rise in the concentration of imidazole in the same pH range did not show any maximum in the activity profile of the 26S proteasome (Figure 4b,c), suggesting that polyamines, in contrast to the other amines, may inhibit the activity of the 26S proteasome at high concentrations. Furthermore, the 25 mM carbonate–phosphate buffer at a ratio of 24:1, imitating physiologically relevant cellular conditions, was supplemented with 10 mM Spm, providing half of the maximal activation rate [49]. Proteasome activity was measured at different pH levels in this buffer, as well as separately in the carbonate–phosphate and Spm buffers (Figure 4d). The addition of polyamines to the carbonate–phosphate buffer preserved the activity of the 26S proteasome at an alkaline pH, suggesting that Spm compensates for carbonate-driven proteasome inhibition.

The activity of the 20S proteasome upon addition of various concentrations of Spm constantly increased and no maximum was observed (Figure 4e). Similar to the 26S proteasome, maximal activation of 20S proteasome was observed at pH 8.0–8.5. Profiling of the activity of 20S proteasome in the carbonate–phosphate buffer supplemented with 10 mM Spm resulted in a skewed distribution, reaching its maximum at pH 7.5 (Figure 4f). The reason for the different activation profiles of the 26S and 20S proteasomes by Spm is not completely clear at present. It is possible that the high concentration of polyamines, together with activation of the 20S catalytic particle, also affects the 19S regulatory particle, and therefore structurally disturbs the diffusion of the substrate into the 20S particle.

A comparison of the ratio of the chymotrypsin-like activity of the 26S proteasome measured in a carbonate–phosphate buffer supplemented with arginine, ornithine, Spd, or Spm to the activity of the 26S proteasome measured in the carbonate–phosphate buffer alone revealed that spermine has the most pronounced ability to counteract carbonate-driven proteasome stalling at an alkaline pH (7.5–8.0) (Figure 4g). The most reasonable explanation for this experimental observation is deprotonation of proteasomes by carbonate ions at an alkaline pH, leading to proteasome inhibition. If we will compare the activity of 20S and 26S in the anionic buffers, one may suggest that 20S is less affected by these buffers and therefore the observed inhibition of the 26S is rather caused by the rearrangement or dissociation of the regulatory subparticle. Spm, as a proton donor, protects proteasomes from deprotonation during simultaneous exposure to Spm and carbonate and thus preserves its activity (Figure 4h). Finally, we tested if Spm activated proteasome in terms of the protein substrates. To this end, we reconstituted the ubiquitination cascade by incubation of the recombinant Ub^vv^-TagGFP2 with E1 and UbcH5c ligases and further added a polyubiquitinated substrate to the 26S proteasome in the presence or absence of Spm at different pH levels (Figure 4i). Our data revealed that the 26S proteasome is inactivated at an alkaline pH, whereas Spm restores its activity toward polyubiquitinated TagGFP2.

## 4. Conclusions

The mechanisms by which tumor cells invade are complex and may be tuned in response to altered environmental conditions [50]. Because of an enhanced glucose metabolism, proton production and excretion are generally increased in cancers [51]. This, combined with poor perfusion, results in an acidic extracellular pH (pHe) that is toxic for normal cells in malignant tumors (pH 6.5–6.9), which is different from that of normal tissue under physiological conditions (pHe 7.2–7.4) [52]. An acidic pHe increases in vitro activity of cathepsin proteinases [15], which are generally believed to be involved in local invasion [14] and tissue remodeling [53,54]. Additionally, an acidic environment increases vascular endothelial growth factor (VEGF)-driven angiogenesis and inhibits the immune response to tumor antigens [55]. Furthermore, cancer cells exposed to a low pH show increased invasion, both in vitro and in vivo [56].

Cellular acidification and alkalization both shift the cellular metabolism in a manner too rapid to be explained by the delayed effects of transcriptional control [57,58,59]. At least part of these shifts can be explained by protonation—the most rapid and reversible post-translational modification of proteins [60]. In this study, we speculate that intracellular alkalization, common for cancer cells, may lead to impairment of proteasome function. Therefore, cancers have to increase the activity of the UPS by utilizing various extensive pathways, e.g., enhanced expression of the proteasome subunits [61]. Consumption of polyamines may represent an alternative *intensive* route to more active proteasomes, which do not require its elevated expression. In summary, polyamines, which are evidently essential for cancer cells in terms of the growth, invasion, and metastasis, may potentially have one more important function linked to the maintenance of proteasome activity in alkaline conditions. Inhibition of polyamine synthesis and its uptake by polyamine transporters [62] may become a clinically relevant and novel therapeutic strategy to selectively stall proteasomes in tumor cells.
